# A New Phenotype in *Candida*-Epithelial Cell Interaction Distinguishes Colonization- versus Vulvovaginal Candidiasis-Associated Strains

**DOI:** 10.1128/mbio.00107-23

**Published:** 2023-03-01

**Authors:** Arianna Sala, Andrea Ardizzoni, Luca Spaggiari, Nikhil Vaidya, Jane van der Schaaf, Cosmeri Rizzato, Claudio Cermelli, Selene Mogavero, Thomas Krüger, Maximilian Himmel, Olaf Kniemeyer, Axel A. Brakhage, Benjamin L. King, Antonella Lupetti, Manola Comar, Francesco de Seta, Arianna Tavanti, Elisabetta Blasi, Robert T. Wheeler, Eva Pericolini

**Affiliations:** a Department of Surgical, Medical, Dental and Morphological Sciences with Interest in Transplant, Oncological and Regenerative Medicine, University of Modena and Reggio Emilia, Modena, Italy; b Clinical and Experimental Medicine PhD Program, University of Modena and Reggio Emilia, Modena, Italy; c Department of Molecular and Biomedical Sciences, University of Maine, Orono, Maine, USA; d Department of Translational Research and of New Technologies in Medicine and Surgery, University of Pisa, Pisa, Italy; e Department of Microbial Pathogenicity Mechanisms, Leibniz Institute for Natural Product Research and Infection Biology-Hans Knöll Institute (HKI), Jena, Germany; f Department of Molecular and Applied Microbiology, Leibniz Institute for Natural Product Research and Infection Biology-Hans Knöll Institute (HKI), Jena, Germany; g Graduate School of Biomedical Sciences and Engineering, University of Maine, Orono, Maine, USA; h Institute for Maternal and Child Health—IRCCS Burlo Garofolo, Trieste, Italy; i Department of Medical, Surgical and Health Sciences, University of Trieste, Trieste, Italy; j Department of Biology, University of Pisa, Pisa, Italy; Duke University

**Keywords:** *Candida albicans*, epithelial cells, host-pathogen interactions, interferons, vulvovaginal candidiasis

## Abstract

Vulvovaginal candidiasis (VVC) affects nearly 3/4 of women during their lifetime, and its symptoms seriously reduce quality of life. Although Candida albicans is a common commensal, it is unknown if VVC results from a switch from a commensal to pathogenic state, if only some strains can cause VVC, and/or if there is displacement of commensal strains with more pathogenic strains. We studied a set of VVC and colonizing C. albicans strains to identify consistent *in vitro* phenotypes associated with one group or the other. We find that the strains do not differ in overall genetic profile or behavior in culture media (i.e., multilocus sequence type [MLST] profile, rate of growth, and filamentation), but they show strikingly different behaviors during their interactions with vaginal epithelial cells. Epithelial infections with VVC-derived strains yielded stronger fungal proliferation and shedding of fungi and epithelial cells. Transcriptome sequencing (RNA-seq) analysis of representative epithelial cell infections with selected pathogenic or commensal isolates identified several differentially activated epithelial signaling pathways, including the integrin, ferroptosis, and type I interferon pathways; the latter has been implicated in damage protection. Strikingly, inhibition of type I interferon signaling selectively increases fungal shedding of strains in the colonizing cohort, suggesting that increased shedding correlates with lower interferon pathway activation. These data suggest that VVC strains may intrinsically have enhanced pathogenic potential via differential elicitation of epithelial responses, including the type I interferon pathway. Therefore, it may eventually be possible to evaluate pathogenic potential *in vitro* to refine VVC diagnosis.

## INTRODUCTION

Candida albicans is an opportunistic fungus that resides commensally in the vaginal tract of healthy women together with beneficial microbes, such as lactobacilli, and/or potential pathogens, such as Gardnerella vaginalis and group B Streptococcus (1, 2). Given the right circumstances, C. albicans is also able to cause acute and recurrent vulvovaginal candidiasis (VVC). VVC is a very common pathology, affecting 70 to 75% of women of child-bearing age at least once in their lifetime (3, 4). About 5 to 8% of these women will suffer from the recurrent form (RVVC), defined as the occurrence of at least four episodes of the acute form every year, requiring continual antifungal therapy (5, 6). Clinical signs of VVC include itching, burning, pain, and redness of the vaginal mucosa, often accompanied by vaginal white discharge, with a drastic reduction in the quality of life and mental well-being of women who are affected by this pathology. Moreover, VVC has been linked to a reduction in fertility (7, 8). Some host conditions have been implicated in VVC onset, although few factors have been associated with higher risk in multiple studies (1, 9–12). In addition, although some fungal traits are required in murine infection models, it is still not clear what induces C. albicans to switch from a harmless commensal to a virulent pathogen that triggers the onset of human VVC infection symptoms.

The current data suggest that in healthy subjects, C. albicans is tolerated in low numbers as a predominantly yeast-form commensal on the mucosal surface without triggering an epithelial immune response (1, 13). When local defense mechanisms are dampened, both C. albicans burden and virulence increase, and this may exceed the tolerance threshold of epithelial cells. This may occur due to estrogen-dependent fungal immune evasion (14). As the host responds and the fungus triggers an intense inflammatory response, this leads to proinflammatory cytokine release (such as interleukin-1β [IL-1β] and IL-6) and recruitment of nonprotective neutrophils (1, 12, 13, 15, 16). Several components have been implicated in the *in vitro* vaginal epithelial response to C. albicans, including upregulation of type I interferon (IFN), changes in many metabolic pathways, enhanced mitochondrial damage, activation of platelet-derived growth factor (PDGF) BB and NEDD9 pathways, and NLRP3 inflammasome activation (12, 17, 18).

Our previous work analyzing samples directly from women showed that there is a strong bias toward hyphal morphology and β-glucan exposure in fungi isolated directly from VVC patients compared to those from colonized women (19). However, it is unknown if this is driven by intrinsic fungal factors and/or host-associated traits. Studies in murine models suggest that there are strain-specific differences in the ability of clinical C. albicans isolates to cause disseminated disease (20), intestinal tract colonization (21–23), oropharyngeal thrush (24, 25), and vulvovaginal infection (26, 27). In addition to variation in VVC virulence, significant heterogeneity in filamentous growth, biofilm formation, candidalysin production, macrophage interaction, and other phenotypes has been described for VVC strains (26, 28–32). Several C. albicans genes have been implicated in inducing inflammation in murine VVC models, including those controlling *in vitro* hyphal growth and production of the candidalysin toxin and secretory aspartyl proteinases (SAPs) (26, 33–35). However, these phenotypes of filamentous growth, biofilm formation, candidalysin production, and SAP expression are found equally in VVC and colonizing strains, which is inconsistent with the idea that any of these easily measured *in vitro* phenotypes is a primary driver of virulence in human infection. Thus, despite years of work it has not yet been possible to identify any *in vitro* phenotypes or disease-causing traits in C. albicans that correlate with human VVC as opposed to asymptomatic colonization. We hypothesized that any VVC virulence trait should be more common in isolates from VVC patients, although present in a subset of isolates from asymptomatic women that are capable of causing disease in the context of the right host environmental perturbation.

Here, we measured both intrinsic fungal traits and fungal traits revealed upon interaction with the host to identify virulence traits associated with symptomatic VVC. We found that the strains do not differ in overall genetic profile or behavior in culture media (i.e., multilocus sequence type [MLST] profile, rate of growth, and filamentation), but they have strikingly divergent behaviors when interacting with vaginal epithelial cells. Specifically, VVC pathogenic isolates grow more prolifically and are shed more from epithelial cells than colonization isolates. Transcriptome sequencing (RNA-seq) analysis of vaginal epithelial cell challenges with a selected VVC strain and colonizing strain revealed many differentially regulated pathways, including the type I interferon, integrin, and ferroptosis pathways. Of particular interest, the type I interferon pathway is activated more by the colonizing strain, and we find that blocking its activity limits strain-specific fungal shedding. Taken together, these data suggest that VVC strains may intrinsically have greater pathogenic potential via differential elicitation of epithelial responses, including through the type I interferon pathway. Therefore, it may be possible to evaluate the pathogenic potential of *Candida* isolates *in vitro* to refine diagnoses and more readily determine if C. albicans is the likely agent for vaginitis symptoms or a harmless commensal.

## RESULTS

### VVC strains do not group phylogenetically.

**FIG 1:**
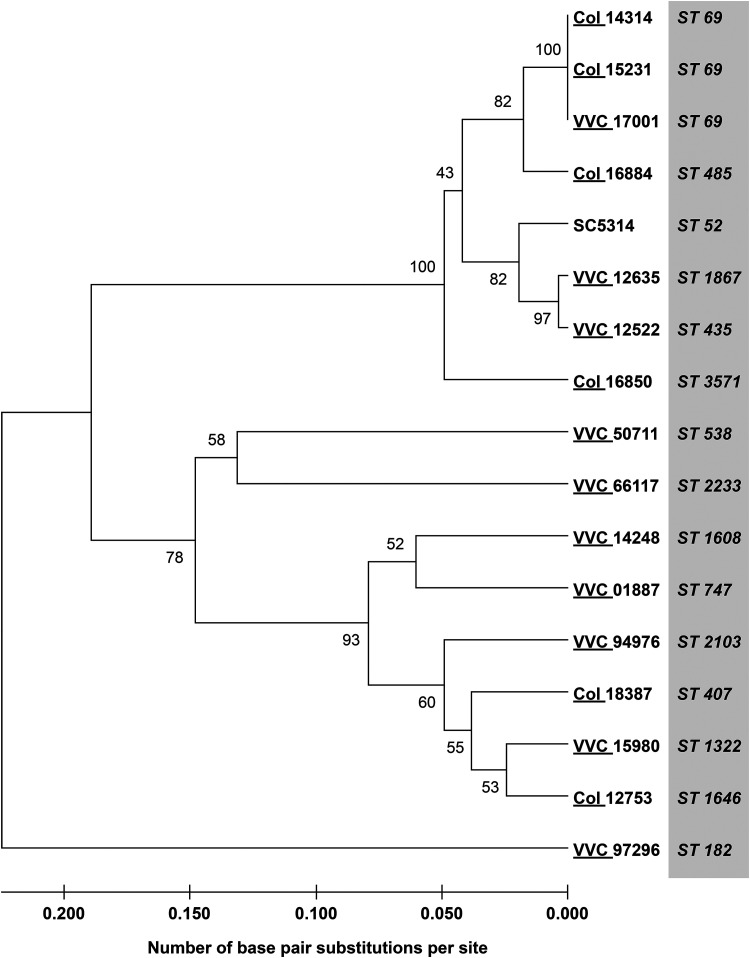
Unrooted neighbor-joining tree showing *p*-distance for C. albicans clinical isolates typed by MLST, using SC5314 as reference strain. The number for each cluster node indicates the bootstrap value. Bootstrapping was set at 500.

To date, it has not been possible to categorize C. albicans strains as “VVC pathogenic” versus “colonizing” through correlation of *in vitro* pathogenic phenotypes with strains originating from VVC. Here, we tested the hypothesis that some C. albicans strains have virulence traits that make them intrinsically more likely to cause symptomatic VVC in healthy women: VVC pathogenic. We took a mixed set of strains that had been directly characterized from vaginal swabs for morphology and cell wall epitope unmasking (19). This study and others have demonstrated that fungi actively causing VVC tend to be in hyphal form, whereas those from asymptomatic women tend to be in yeast form when examined directly in vaginal swabs. We first tested if any C. albicans clades are more associated with VVC by MLST analysis. MLST analysis was performed according to established protocols and did not reveal any genetic clustering distinguishing strains causing VVC versus those involved in colonization (Fig. 1). C. albicans isolates fell into different clades (with a majority of clade I strains [data not shown]).

### VVC strains do not group based on candidalysin allele or expression.

**FIG 2:**
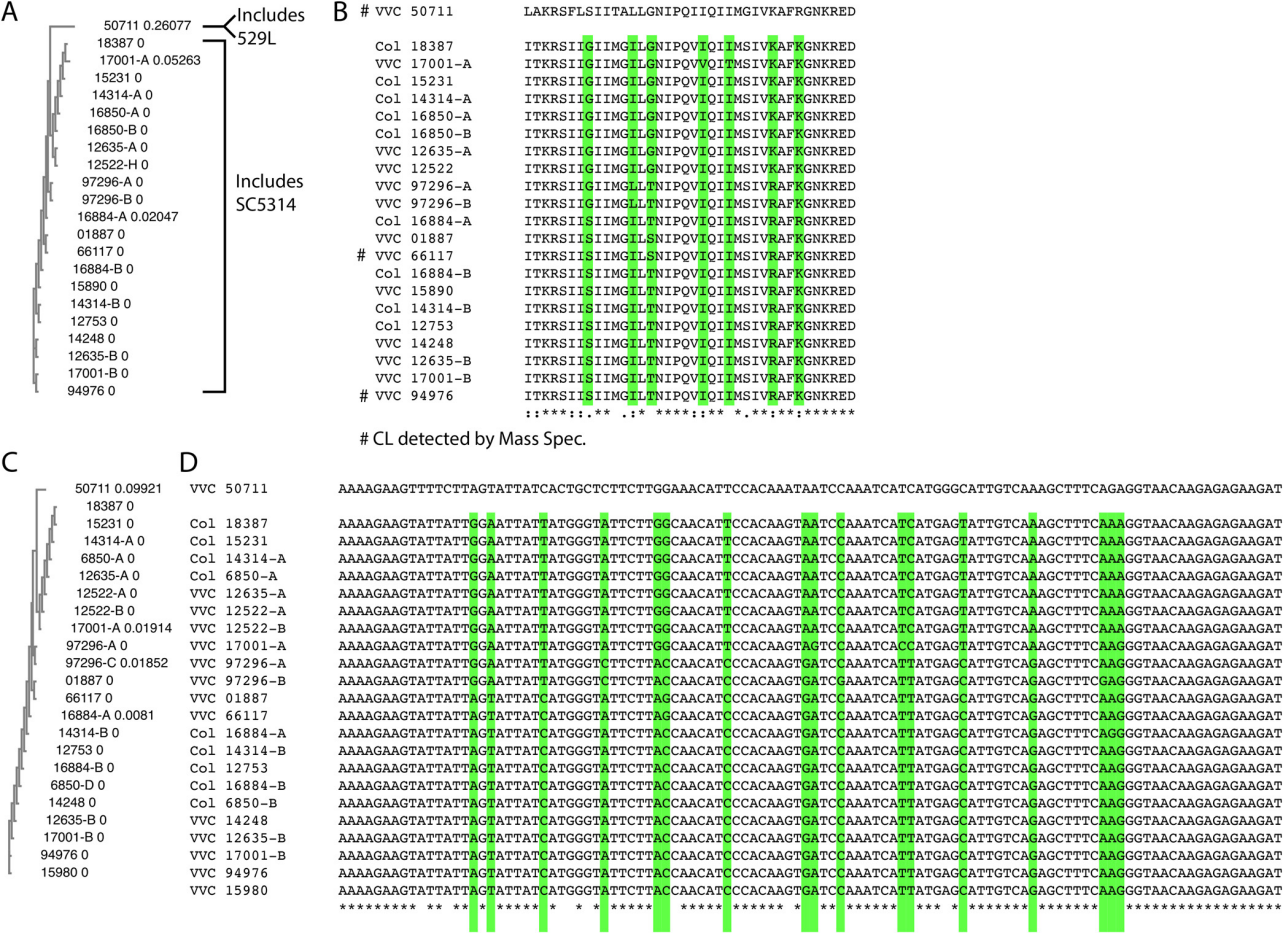
Sequence comparisons of candidalysin sequences at the protein and DNA levels from vaginal isolates. (A) Cladogram of aligned protein sequences with relative distances by nearest-neighbor joining. The 50711 sequence groups away from all the other sequences and matches with the sequence from the 529L clinical isolate. (B) Peptide alignment. Highlighted in green are positions with variability even within the SC5314-type allele group. Conservation among the 22 individual alleles is indicated as fully conserved (*), conservative (:), semiconservative (.), or nonconservative (blank) below the alignment. Protein alleles marked with a # to the left indicate those where candidalysin was detected *in vitro* by proteomics. (C) Cladogram of aligned nucleotide sequences with relative distances by nearest-neighbor joining; (D) nucleotide alignment. Highlighted in green are positions with variability even within the SC5314-type allele group. Fully conserved positions are denoted with asterisks below the alignment. Alleles are indicated by strain number and then -A and -B to denote variable alleles in a single strain. “Col” indicates a strain from an asymptomatic individual, and “VVC” denotes a strain from a VVC patient.

It has been recently shown that candidalysin, a C. albicans hypha-associated toxin, is important for inducing an inflammatory signal in a murine VVC model, suggesting a potentially pathogenic role during VVC (36). Furthermore, the two main types of candidalysin alleles in clinical isolates (SC5314- or 529L-like) have quite different abilities to elicit inflammation in the murine model of VVC (26). Clinical VVC isolates have either the SC5314 or 529L allele of candidalysin in approximately even ratios, raising the question of whether these allelic differences are important for VVC pathogenesis in humans (26). We sought to test, in our collection of clinical *Candida* strains, whether there was any association between candidalysin genotype or expression and symptomatic VVC. The candidalysin-encoding region of *ECE1* was amplified from each isolate, and the DNA sequences of alleles from individual isolates were aligned. Many strains (8/16 at the DNA level and 6/16 at the amino acid level) were found to be heterozygous at the *ECE1* locus in the region encoding candidalysin, although even those heterozygous strains had very similar candidalysin sequences for both alleles at both the DNA (>87% identity) and predicted protein (>89% identity) levels (Fig. 2). All but one of the alleles grouped with the SC5314 sequence, while the outlier grouped with the 529L sequence (Fig. 2A; see Fig. S1 in the supplemental material). Interestingly, the relationships according to MLST (Fig. 1) did not correspond well to the relationships according to candidalysin *ECE1* sequences (Fig. 2). Accordingly, in this set of clinical isolates there was no association between candidalysin sequence and symptomatic VVC infection (Fig. 2).

10.1128/mbio.00107-23.1 FIG S1 Sequence comparisons of candidalysin from vaginal isolates compared to those obtained from 23 other clinical isolates available in NCBI. (A) Cladogram of aligned protein sequences with relative distances; (B) peptide alignment. Highlighted in green are positions with variability even within the SC5314-type allele group. Fully conserved positions are denoted with asterisks below the alignment. Alleles are indicated by strain number and then -A and -B to denote variable alleles in a single strain. The 529L-like clade is outlined with the red boxes. Download FIG S1, PDF file, 0.6 MB. Copyright © 2023 Sala et al. 2023 Sala et al. https://creativecommons.org/licenses/by/4.0/ This content is distributed under the terms of the Creative Commons Attribution 4.0 International license.

Since candidalysin genotype is only one indicator of expression and function, it remains possible that mRNA or protein expression levels of mature candidalysin distinguish colonizing from symptom-inducing strains. Therefore, we measured secreted candidalysin levels from each strain growing *in vitro* under strong hypha-inducing conditions by liquid chromatography-tandem mass spectrometry (LC-MS/MS) as previously described (37). Three clinical isolates (66117, 94976, and 50711) produced detectable levels of candidalysin, but most of the strains failed to produce sufficient levels of candidalysin for detection (indicated by # in Fig. 2B; Data Set S1). There was no significant correlation between lack of detectable levels and isolation from asymptomatic individuals (Fisher’s exact test; *P* = 0.2286). Interestingly, the only three vaginal isolates producing detectable levels were from VVC patients, including the virulent strain with the 529L-like allele (CA50711).

10.1128/mbio.00107-23.4 DATA SET S1 Full data from all proteomics experiments. Supernatants were analyzed as described in Materials and Methods. Download Data Set S1, XLSX file, 0.1 MB. Copyright © 2023 Sala et al. 2023 Sala et al. https://creativecommons.org/licenses/by/4.0/ This content is distributed under the terms of the Creative Commons Attribution 4.0 International license.

### VVC strains do not have greater hyphal growth *in vitro*.

**FIG 3:**
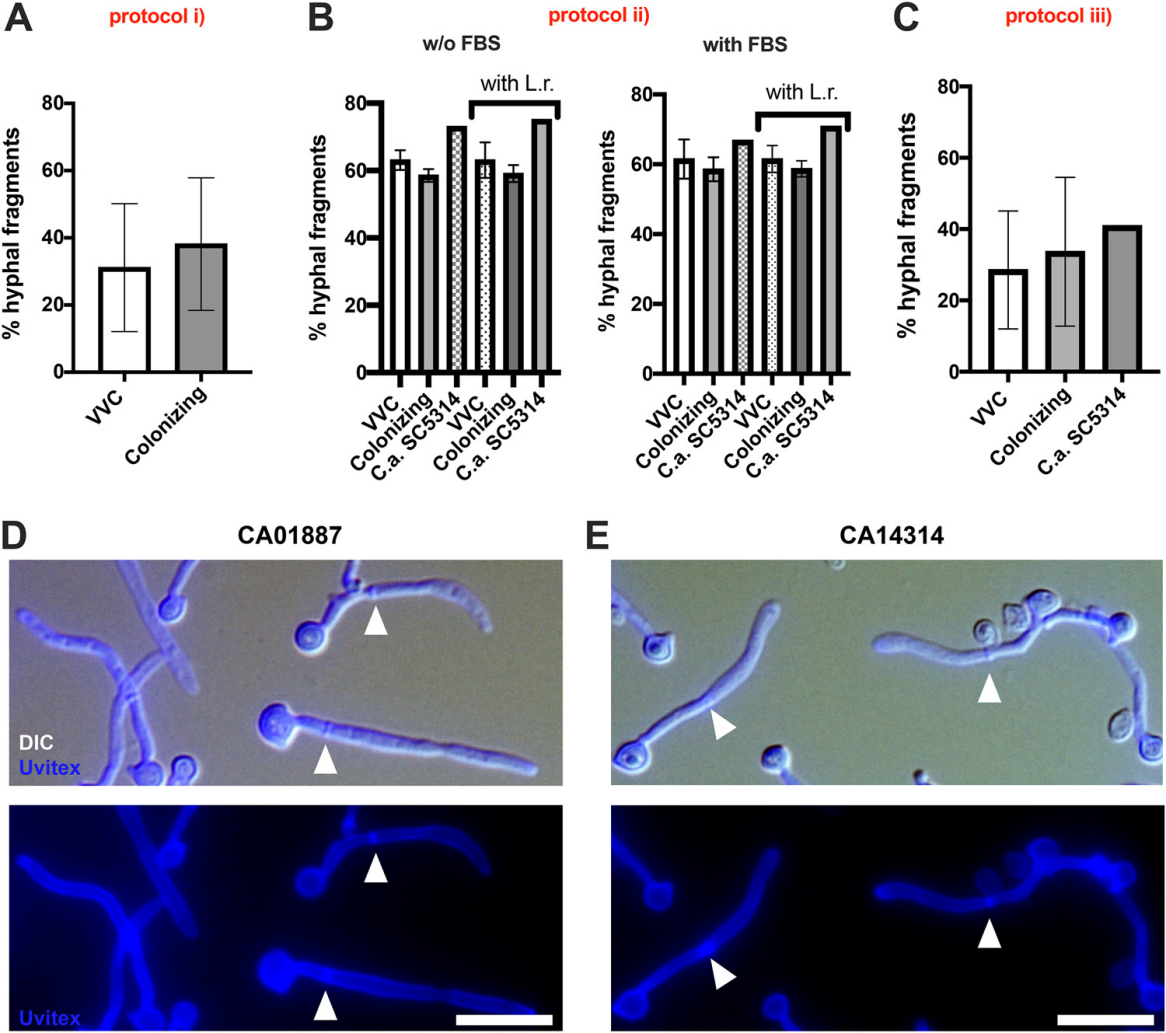
C. albicans hyphal fragment production. Data in the graphs show the mean percentage ± standard deviation (SD) of hyphal fragments produced by VVC and colonizing C. albicans strains after 24 h of culture in RPMI 1640 (protocol i) (A), in RPMI or RPMI plus 10% FCS in the presence or absence of Lactobacillus rhamnosus (L.r.) (protocol ii) (B), or during infection of a monolayer of A431 cells (protocol iii) (C). Statistical analysis was performed according to Student's *t* test (A and C) and Kruskal-Wallis test, followed by Dunn’s multiple-comparison test or one-way ANOVA, followed by Tukey’s multiple-comparison test (left and right graphs of panel B, respectively). Panels D and E show representative images of hyphal segments from the 01887 (D) and 14314 (E) strains from experimental protocol ii, grown in sgRPMI. Arrowheads indicate the septa separating one hyphal segment from another. The data come from at least 3 biological replicates.

In both clinical and murine infection studies, hyphae are associated with C. albicans VVC (19, 38, 39). We sought to find *in vitro* conditions under which VVC strains produce high levels of hyphae and colonizing strains are present largely as yeast, as seen clinically (19). First, we simply quantified filamentous growth after overnight growth in RPMI 1640 tissue culture medium (protocol i). In these assays, all strains produced extensive hyphae and there was no significant difference between strains from colonized versus symptomatic individuals (Fig. 3A). We then tested whether coculture with lactobacilli might selectively reduce hyphal growth in colonizing strains, as these bacteria are often present in the vaginal environment and can inhibit morphological transitions of C. albicans (40–42). We grew C. albicans strains for 3 h in RPMI 1640 or RPMI 1640 plus 10% fetal bovine serum (FBS) in the presence or absence of Lactobacillus rhamnosus and then quantified their morphology (protocol ii). Again, there were no significant differences in hyphal formation between strains from VVC women versus those from colonized women (Fig. 3A and B; see Table S1A in Data Set S2). At the same time, although different growth rates may have occurred, no differences in overall growth were found after culture for 24 h in YPD broth (Fig. S2A). These results suggest that each of these strains is able to make sufficient hyphae in tissue culture medium to be pathogenic and that unidentified environmental factors in the host may play a large role in limiting filamentous growth during colonization as opposed to infection.

10.1128/mbio.00107-23.2 FIG S2 C. albicans growth in YPD medium and adhesion on abiotic surfaces. (A) Data in the graph show the mean ± standard error of the mean (SEM) in CFU per milliliter from five different experiments after 24 h of growth of VVC and colonizing C. albicans strains in YPD broth at 37°C. (B) *Candida* adhesion to plastic was analyzed by crystal violet staining. Data in the graph show the mean OD_570_ ± SEM of triplicate samples from 2 different experiments. The dotted line corresponding to the OD value of 0.182 indicates the background signal of the PBS. Data were analyzed by unpaired Student’s *t* test. Download FIG S2, PDF file, 0.5 MB. Copyright © 2023 Sala et al. 2023 Sala et al. https://creativecommons.org/licenses/by/4.0/ This content is distributed under the terms of the Creative Commons Attribution 4.0 International license.

10.1128/mbio.00107-23.5 DATA SET S2 Supplemental tables. Table S1A shows full data from hyphal induction experiments. Fungi were grown and hyphae were counted as described in Materials and Methods. Table S1B presents exact DESeq2 values for the type I interferon, integrin, and ferroptosis pathways. For experimental and methodology details, see Fig. 6 and Materials and Methods. Table S1C shows fungal strains and sources. Table S1D shows a description of data taken along with vaginal samples yielding new strains from Trieste. Download Data Set S2, XLSX file, 0.02 MB. Copyright © 2023 Sala et al. 2023 Sala et al. https://creativecommons.org/licenses/by/4.0/ This content is distributed under the terms of the Creative Commons Attribution 4.0 International license.

Since vaginal epithelial cells can influence C. albicans growth *in vitro* (43), we examined if differences in filamentation between these two groups of isolates were associated with interaction with host cells. We challenged A431 vaginal cells for 24 h with C. albicans strains and then measured the propensity of C. albicans strains to form hyphae (protocol iii). Again, no cohort-specific differences in the percentage of hyphal segments were observed (Fig. 3C; Table S1A in Data Set S2). Taken together, we found no differences in overall filamentation between cohorts of strains isolated from VVC or colonized women, even in the context of infection of the vaginal epithelium. This suggests that more complex host intrinsic mechanisms may play the most important role in regulation of filamentous growth and pathogenesis in human vaginal mucosa (1, 44).

### VVC strains proliferate more and are shed more during epithelial infection.

**FIG 4:**
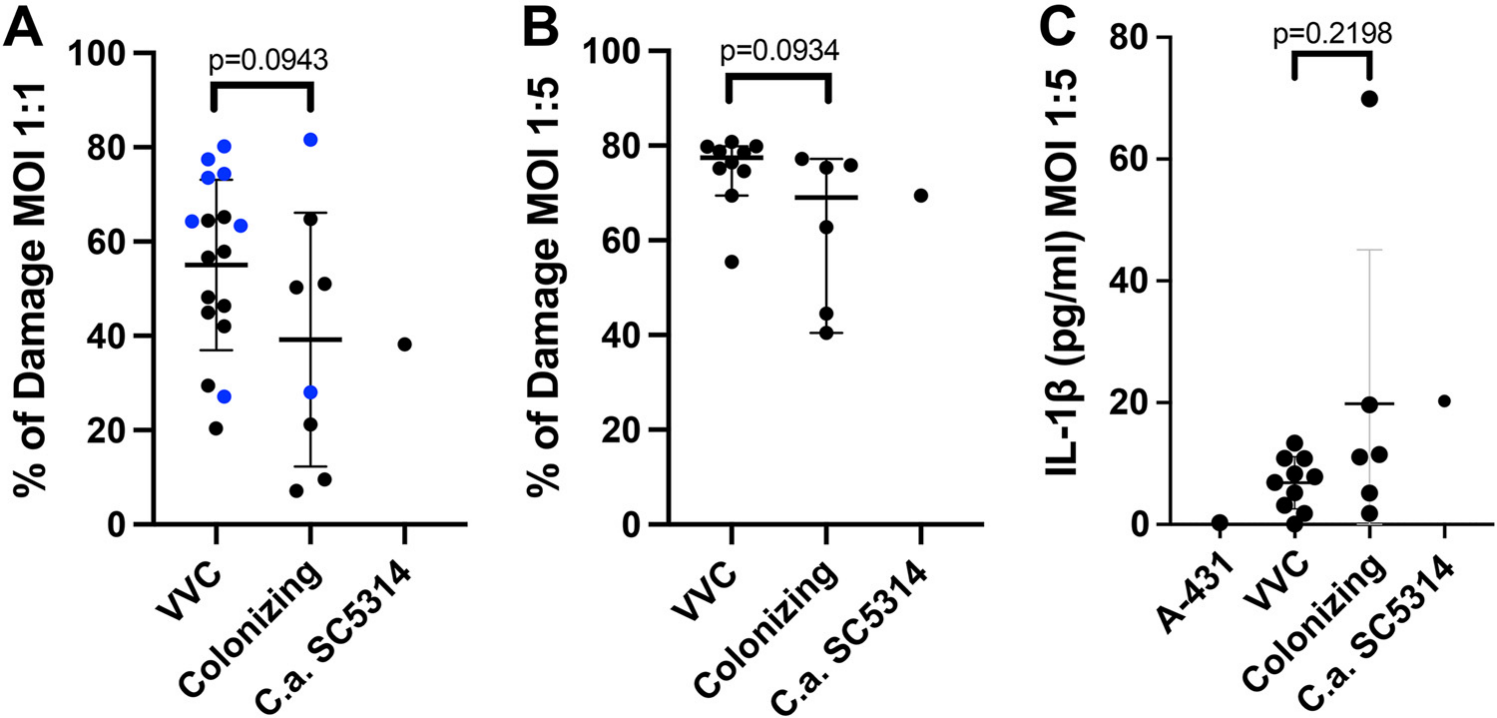
Vaginal epithelial cell damage and IL-1β production. (A and B) Vaginal cell damage (LDH release) after 24 h of infection with VVC and colonizing C. albicans strains or by the reference strain SC5314 at an MOI of 1:1 (A) or 1:5 (B). The blue dots highlighted in panel A indicate results from an additional group of 9 isolates the provenance of which had been previously reported (see reference 44). (A) Mean ± SD of each cohort; (B) median of each cohort with 95% confidence interval (CI). Each data point represents the average from three biological replicates. (C) IL-1β (picograms per milliliter) released by vaginal cells after 24 h of infection with VVC and colonizing C. albicans strains or by the reference strain SC5314 at an MOI of 1:5; (C) mean ± SD of each cohort. Each data point represents the average from 4 biological replicate experiments. Statistical analysis was performed according to unpaired Student's *t* test (A; MOI of 1:1) and Mann-Whitney test (B and C; MOI of 1:5), according to data normality. *P* values of >0.05 were considered not significant and are shown above the data.

VVC is associated with inflammation, pain, and vaginal discharge (3, 4). We sought to translate these human disease symptoms into *in vitro* phenotypes and test for differences that might distinguish colonizing isolates from symptomatic isolates. First, we assessed the ability of each strain to damage vaginal epithelial cells, resulting in release of lactate dehydrogenase. We challenged a monolayer of A431 cells with each C. albicans isolate and measured lactate dehydrogenase (LDH) release. Here, we observed no significant difference in LDH release provoked by VVC versus colonizing strains. Nonetheless, there was a weak trend toward reduced epithelial damage caused by colonizing strains (irrespective of the multiplicity of infection [MOI] used), compared to VVC pathogenic strains (Fig. 4A and B). Interestingly, this trend holds for an independent set of mixed pathogenic/colonizing isolates from vaginal swabs described by Ardizzoni et al. (44) (blue dots in Fig. 4A).

Next, we assayed epithelial cell-produced inflammatory signals. It is known that IL-1β and polymorphonuclear leukocyte (PMN) recruitment are associated with symptomatic inflammation characteristic of VVC in humans (19, 39, 45). We measured levels of a suite of secreted inflammatory factors and found overall low elicitation from A431 cells, similar to what has been reported (36, 46). While some IL-1β was produced by epithelial cells upon C. albicans challenge, IL-1β levels in cell culture supernatants were not significantly different between strains from VVC versus colonized women (Fig. 4C).

Finally, we sought an *in vitro* technique to approximate the process leading to vaginal discharge, which contains epithelial cells and associated microbes being shed into the vaginal lumen (47). To maintain tissue homeostasis, the vaginal epithelium induces shedding of mature superficial epithelial cells, while the renewal of cells conserves barrier integrity (48, 49). Bacterial infection can induce the exfoliation of epithelial cells (50–53). C. albicans is shed in association with host cells, providing a potential mechanism for elimination of invading hyphae but also potentially damaging the epithelium (54).

**FIG 5:**
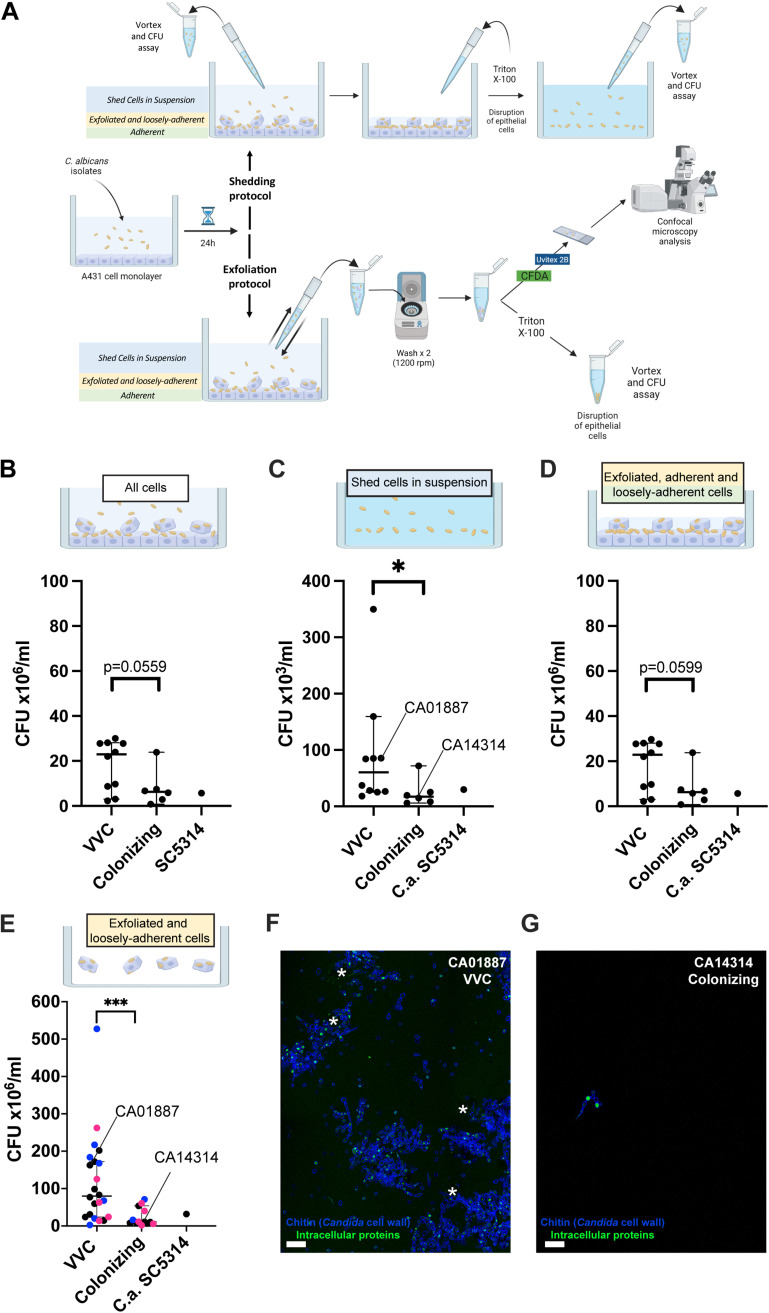
C. albicans shedding from vaginal epithelium. (A) Shedding of epithelial and fungal cells was measured by either the shedding protocol (A, upper) or the exfoliation protocol (A, lower). Epithelial or fungal cells could be attached to the substrate, loosely attached to the substrate, or shed in suspension. Each protocol measures different fractions of the total challenge, as indicated by the workflow. The shedding protocol measures shed cells in suspension (top panels), whereas the exfoliation protocol measures shed cells bound to exfoliated cells and/or loosely associated with the epithelium (bottom panels). (B to G) Data in the graphs show the mean ± SD CFU from 3 different experiments after 24 h of infection of the vaginal epithelial monolayer with the indicated C. albicans strains at an MOI of 1:1. (B to D) Shedding protocol. (B) Overall C. albicans CFU for each infection (all cells); (C) C. albicans CFU shed in the supernatants (shed cells in suspension), and (D) C. albicans CFU attached to or loosely associated with vaginal epithelium (exfoliated, adherent, and loosely adherent cells). (E to G) Exfoliation protocol. (E) C. albicans CFU shed in supernatant in association with shed epithelial cells (exfoliated and loosely adherent cells). In panel E, the black dots represent the standard 16 clinical strains used throughout the study, blue dots indicate an additional group of 9 isolates tested (see reference 44), and magenta dots indicate a third group of 10 isolates from women with RVVC or healthy colonized women obtained from IRCSS Burlo Garofolo Trieste, Italy. Statistical analysis was performed according to the Mann-Whitney test. *P* values of <0.05 (*) and <0.001 (***) were considered significant. *P* values of >0.05 are not considered significant and are shown above the data. Panels F and G show representative images of C. albicans shedding of the VVC strain CA01887 (F) and the colonizing strain CA14314 (G). Exfoliated and loosely adherent cells are shown, including epithelial cells with punctate CFDA in the cytoplasm (marked by asterisks) and *Candida* cells (blue with bright dots of CFDA staining). Scale bar = 20 μm.

We employed two similar experimental protocols to measure the level of C. albicans shed from an infected epithelium. First, we used the shedding protocol described by Graf et al. (54) to quantify the total level of C. albicans shed in the supernatants (shed cells in suspension, as shown by the blue fraction in Fig. 5A) and the live C. albicans cells associated with the epithelium (exfoliated and loosely adherent cells, as shown by the yellow fraction in Fig. 5A, plus adherent cells, as shown by the green fraction in Fig. 5A). The cell types and measurements of this shedding protocol are summarized in the upper part of Fig. 5A. We measured overall fungal growth on vaginal epithelia (all cells) and found a trend that VVC strains proliferate more robustly than colonizing strains (*P* = 0.0559) (Fig. 5B). We then measured shedding and found a significantly higher number of CFU in supernatants from challenges with VVC C. albicans strains than in those with colonizing isolates (Fig. 5C; shed cells in suspension; *P* = 0.0110). In contrast, no significant differences were observed in the level of C. albicans attached to the epithelium between strains from the VVC versus colonizing group (Fig. 5D; exfoliated, adherent, and loosely adherent cells), although there was a trend toward fewer fungal cells in the colonizing strain cohort (*P* = 0.0599). Unfortunately, this protocol does not examine if epithelial cells remain adherent or exfoliate from the substrate, nor does it quantify fungal cells associated with exfoliated epithelial cells.

To measure the level of *Candida* associated with nonadherent/exfoliated epithelial cells, we developed the exfoliation protocol (summarized in the lower part of Fig. 5A). By gently pipetting off nonadherent epithelial and fungal cells from the epithelial monolayer, we were able to measure live C. albicans cells that were either nonadherent or were shed in association with exfoliated epithelium (exfoliated and loosely adherent cells, shown as yellow and green in Fig. 5A). Similar to the shedding protocol, we also found significantly higher levels of fungal shedding in challenges with VVC versus colonizing strains (*P* = 0.0003) (Fig. 5E; exfoliated and loosely adherent cells). Confocal microscopy analysis of supernatants from representative VVC (CA01887) and colonizing (CA14314) strains revealed a correspondingly higher level of epithelial cells shed in association with live *Candida* after CA01887 infection than in the CA14314 strain infection (Fig. 5F to G). Interestingly, shed fungal cells in VVC isolate challenges were often found associated with vaginal epithelial cells (marked with asterisks), suggesting that the fungal shedding could be due to exfoliation of epithelial cells. To determine if differential adhesion to plastic accounted for any cohort-specific differences in shedding, the assay was carried out without any preaddition of epithelial cells. There was no overall difference in plastic adhesion between cohorts of isolates, suggesting that shedding differences are not mediated by fungus-plastic interaction (Fig. S2B). Taken together, these complementary results suggest that the VVC-associated isolates induce more shedding of *Candida* and exfoliation of epithelial cells than colonization-associated isolates. This is consistent with the idea that these VVC-associated strains induce more vaginal discharge in women, leading to symptomatic disease.

### Differential epithelial response to VVC versus colonizing strain.

**FIG 6:**
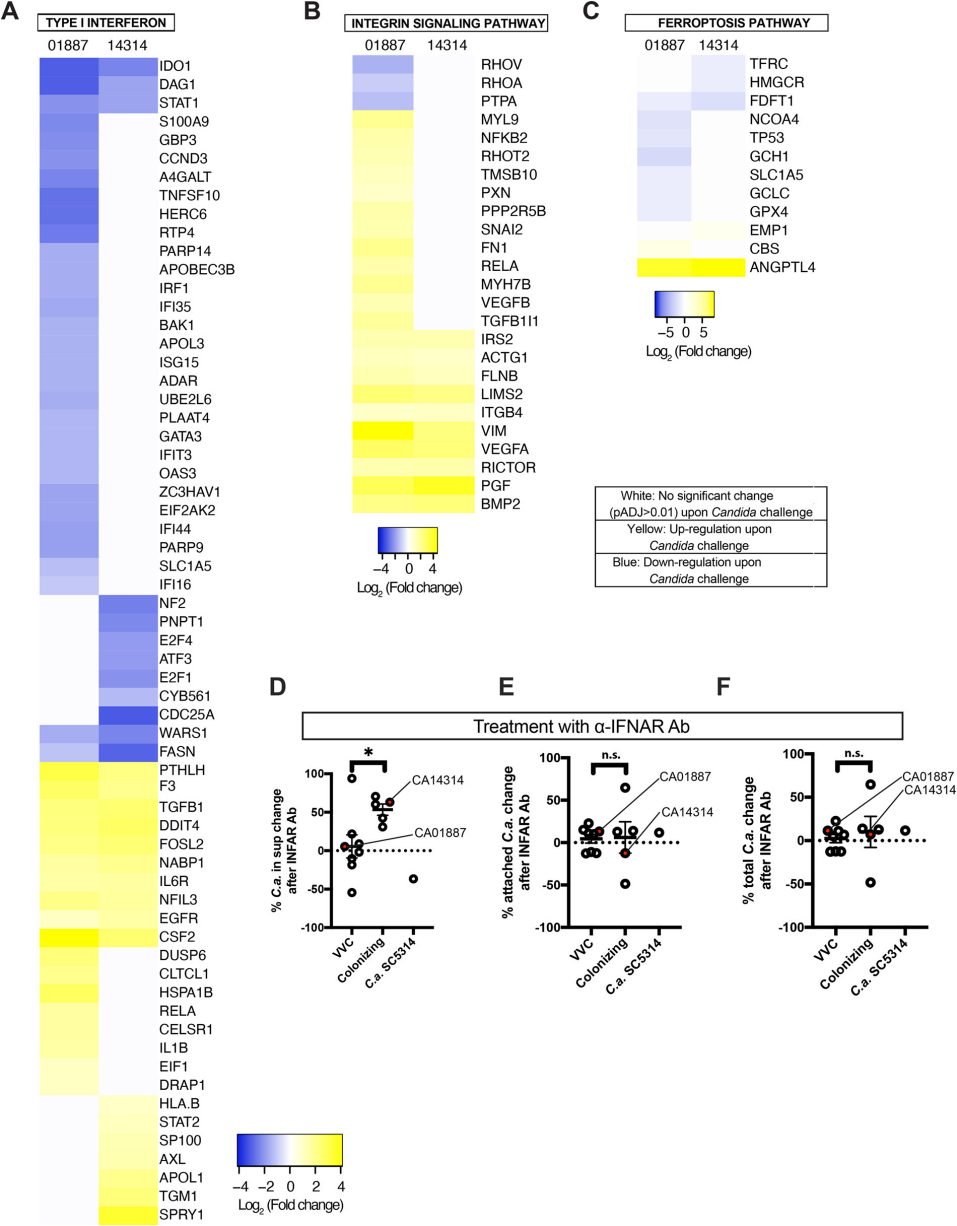
CA01887 and CA14314 differentially activate type I interferon, integrin, and ferroptosis pathways. A431 vaginal epithelial cells were challenged with either CA01887 or CA14314 for 24 h. Differential RNA-seq was performed on two biological replicates of mock-infected versus infected challenges and analyzed with DEseq2 and Ingenuity Pathway Analysis. (A to C) Differentially regulated genes from three pathways with divergent responses upon C. albicans challenge are shown with statistically significant upregulation (yellow), significant downregulation (blue), or no significant change (white). The range for each is shown below (values are the log_2_ ratio of fungal challenge to mock challenge). The cutoff for significant changes was an adjusted *P* value (*P*_adj_) of <0.01. (D to F) Summary of IFNAR inhibition of shedding for several VVC and colonizing strains. Shown is the mean percentage of change ± SD in CFU from at least 3 different experiments after 24 h of infection of the vaginal epithelial monolayer with VVC or colonizing strains in the presence or absence of neutralizing IFNAR antibody (Ab). All experiments used the shedding protocol. (D) C. albicans CFU shed in the supernatants (cells in suspension); (E) C. albicans CFU attached to vaginal epithelium (exfoliated, adherent, and loosely adherent cells); (F) overall C. albicans CFU for each infection (all cells). Red dots indicate the strains CA01887 and CA14314, which were used in the original RNA-seq experiments. The statistical comparisons between VVC and colonizing strains were performed according to the unpaired Student's *t* test. *P* values of <0.05 (*) were considered significant. n.s., not significant (*P* > 0.05).

To discover the reasons for this differential behavior of C. albicans isolates in the shedding process, we looked more carefully at epithelial responses to the two classes of C. albicans strains, performing RNA-seq analysis. We challenged a monolayer of A431 vaginal epithelial cells with a representative VVC strain or a colonizing strain (i.e., CA01887 and CA14314, respectively [data on these strains are shown in Fig. 5]) and isolated RNA from the infected cells. Several different human signaling pathways were significantly associated with C. albicans challenge for each of the two strains, based on Ingenuity Pathway Analysis (IPA) (Fig. S3 and Data Set S3). However, there were also pathways with divergent activation upon infection with VVC and colonizing strains, including the type I interferon (Fig. 6A), integrin (Fig. 6B), and ferroptosis pathways (Fig. 6C; Table S1B in Data Set S2) signaling pathways. These three pathways were selected for more detailed IPA analysis due to published links between the pathways and pathogen challenge or infection (52, 53, 55–62). More detailed analysis of pathways indicated that the VVC-associated strain evoked a weaker type I IFN and ferroptosis pathway induction but a stronger integrin pathway activation. Gene Ontology (GO) enrichment analysis identified hypoxia signaling as regulated in both infections, which is a GO biological process term previously identified by transcriptomic analysis of *Candida*-A431 challenges (63) (Data Set S4). While the roles of hypoxia, integrin signaling, and ferroptosis were not investigated further, we went on to functionally test the role of type I interferon signaling in fungal shedding because of its known role in A431 epithelial response to C. albicans.

10.1128/mbio.00107-23.3 FIG S3 Complete Ingenuity Pathway Analysis data for CA01887 and CA14314 RNA-seq challenge experiments. All differentially regulated pathways are shown. Download FIG S3, TIF file, 7.2 MB. Copyright © 2023 Sala et al. 2023 Sala et al. https://creativecommons.org/licenses/by/4.0/ This content is distributed under the terms of the Creative Commons Attribution 4.0 International license.

10.1128/mbio.00107-23.6 DATA SET S3 Ingenuity Pathways Analysis (IPA) with canonical pathway enrichment analysis. IPA canonical pathways annotated to lists of differentially expressed genes for the three pairwise sample group comparisons were analyzed and are shown in separate worksheets. Each worksheet lists the pathway name, probability [−log(*P* value)], ratio of differentially expressed genes to total number of genes in pathway, Z-score indicating predicted change in pathway activity activation (positive) or repression (negative) based on gene expression changes, and the symbols of differentially expressed genes that mapped to the pathway. For the selection of differentially expressed genes used in the analysis, an FDR threshold of <0.01 was used for the CA01887-infected and CA14314-infected comparisons to mock-infected A431 cells, and an FDR threshold of <0.05 was used for the CA01887-infected and CA14314-infected comparison. The three lists of differentially expressed genes used are provided in Data Set S5 and Data Set S6. Download Data Set S3, XLSX file, 0.1 MB. Copyright © 2023 Sala et al. 2023 Sala et al. https://creativecommons.org/licenses/by/4.0/ This content is distributed under the terms of the Creative Commons Attribution 4.0 International license.

10.1128/mbio.00107-23.7 DATA SET S4 Gene Ontology (GO) biological process enrichment analysis. GO biological process terms annotated to lists of differentially expressed genes for the three pairwise sample group comparisons were analyzed using PantherDB and DAVID. The three lists of differentially expressed genes, background genes, and results of the PantherDB and DAVID analyses are shown in separate worksheets. For the selection of differentially expressed genes, an FDR threshold of <0.01 was used for the CA01887-infected and CA14314-infected comparisons to mock-infected A431 cells, and an FDR threshold of <0.05 was used for the CA01887-infected versus CA14314-infected comparison. The PantherDB overrepresentation analysis was done by analyzing the PantherDB GO-Slim biological process terms and the background genes provided in the worksheet. The DAVID enrichment analysis was performed using DAVID GO biological process FAT terms. The three worksheets that list the DAVID analysis results have these columns: GO biological process FAT term, number of differentially expressed genes annotated to the term, percentage of differentially expressed genes annotated to term, unadjusted enrichment *P* value, list of Ensembl gene identifiers for differentially expressed genes annotated to the term, total number of differentially expressed genes, number of background genes annotated to term, total number of background genes, fold enrichment, Bonferroni adjusted *P* value (padj), Benjamini-Hochberg adjusted *P* value, and FDR-adjusted *P* value. The three worksheets that list the PantherDB analysis results have these columns: GO-Slim biological process term, number of genes from the background annotated to the term, number of differentially expressed genes annotated to the term, the number of expected genes annotated to the term, fold enrichment, unadjusted Fisher's exact test *P* value, and FDR-adjusted *P* value. GO analysis by biological process was carried out with Panther (http://www.pantherdb.org) or DAVID (https://david.ncifcrf.gov/). Download Data Set S4, XLSX file, 1.3 MB. Copyright © 2023 Sala et al. 2023 Sala et al. https://creativecommons.org/licenses/by/4.0/ This content is distributed under the terms of the Creative Commons Attribution 4.0 International license.

### Differential type I interferon pathway activation functionally linked to shedding difference.

Lower type I interferon pathway induction suggested the possibility that VVC-causing C. albicans strains do not elicit type I interferon when interacting with the host vaginal mucosa, preventing a protective response and allowing greater damage (63). A prediction of this idea is that inhibition of the IFN-α/β receptor (IFNAR) would increase shedding only during challenges with the colonizing strain CA14314. To test this, we used a neutralizing anti-IFNAR antibody and measured shedding with either the colonizing or pathogenic strain (using the shedding protocol). In agreement with this hypothesis, blocking the IFNAR led to significantly increased C. albicans shedding of the colonizing strain but not of the VVC pathogenic strain (Fig. 6D, red dots), while this intervention had no significant effect on the number of viable adherent or total *Candida* cells for either infection (Fig. 6E and F, red dots). By extending these experiments to assay several additional strains, we found overall that the shedding of colonizing strains has greater dependence on IFNAR as opposed to that of the VVC strains, although there is significant heterogeneity in both cohorts (Fig. 6D). Again, IFNAR blockade did not affect viable adherent or total numbers of *Candida* cells (Fig. 6E and F).

## DISCUSSION

Despite decades of work on *in vitro* characterization of vaginal clinical isolates of C. albicans, it has not been possible to attribute any pathogenesis-associated phenotypes that distinguish VVC pathogenic strains from those associated with asymptomatic colonization. This negative evidence has led to a focus on host-specific differences in response to fungi in the vaginal mucosa (1, 12, 13). In contrast, our results distinguish a cohort of VVC isolates from their colonizing counterparts using phenotypes revealed only upon their interaction with vaginal epithelial cells. These data suggest that VVC pathogenic strains are better able to proliferate during epithelial challenge and are shed more efficiently with epithelial cells. These phenotypes are consistent with the frequent presence of white vaginal discharge in VVC, which contains epithelial and fungal cells.

As predicted by the host-microbe damage response framework (13), there are likely both host- and *Candida*-associated conditions that combine to result in an outcome of colonization or symptomatic VVC. Our combined RNA-seq–IFNAR inhibition results with two representative isolates suggest that some colonizing strains may preferentially induce type I interferon, which then aids in epithelial defense and limits shedding and exfoliation. This suggests that VVC pathogenic strains may have the ability to avoid eliciting type I interferon pathway activation and thereby block this protective response in epithelial cells. Type I interferon has not been previously implicated in human VVC by genome-wide association analysis, but recombinant interferon beta has some protective effects in a rat VVC model and type I interferon is present in vaginal fluid of VVC patients (64, 65). Recent work suggests that the C. albicans SC5314 activates the epithelial interferon pathway through the candidalysin toxin, which is linked to mitochondrial damage (63). No secreted candidalysin was detected from either of the strains used in our RNA-seq experiments, although it may be present at higher levels in the invasion pocket during infection (66). While *ECE1* allelic differences are associated with inflammation in murine VVC (26), we found no association between the *ECE1* allele and VVC, suggesting the possibility that candidalysin is more important for different classes of strains (VVC versus colonizing) and/or is more important for murine than human VVC (38).

In addition to differential regulation of type I interferon signaling, IPA identified many other pathways that are differentially induced by the VVC pathogenic strain. One pathway activated only by the VVC strain is the integrin signaling pathway, which is targeted by at least two bacterial pathogens of the vaginal tract: group B Streptococcus and Neisseria gonorrhoeae (52, 53). C. albicans encodes proteins that engage with integrins, in addition to having an integrin-like cell wall protein of its own (56–58, 60, 61, 67). Another pathway predicted to be activated preferentially by the colonizing strain was the ferroptosis pathway, a cell death pathway that is also targeted by bacterial pathogens (55, 59, 62). Intriguingly, iron chelators reduce C. albicans virulence in both oral and vaginal murine disease models, although this might be attributable to a direct antifungal effect as well (68, 69). To date, there have been no reports of either integrin or ferroptotic signaling being involved in VVC, although recent work implicates immune cell ferroptosis in *Candida* virulence (70).

Vaginal isolates of C. albicans have been analyzed both genetically and phenotypically *in vitro* by many groups, although no clades have been linked to symptomatic VVC in multiple locations. These studies, together with the current report, indicate that C. albicans VVC isolates come from all clades and express heterogeneity with respect to biofilm production, macrophage interactions, and hyphal induction under different conditions (19, 26, 28–32). However, none of these phenotypes has been specifically linked to VVC strains versus colonizing strains in human disease.

Taken together, our data suggest that VVC strains may have more intrinsic pathogenic potential via differential elicitation of epithelial responses, including through the type I interferon pathway. Therefore, it may be possible to evaluate pathogenic potential by shedding and exfoliation *in vitro* to refine diagnoses and more readily determine if C. albicans is the likely agent for vaginitis symptoms. It is important to note that our strains from colonized and VVC individuals exhibit diverse levels of shedding with overlapping ranges, suggesting they are not homogeneous. It is likely that some strains isolated from non-symptomatic patients nevertheless have the ability to cause VVC under the right circumstances and thus should be considered “VVC” strains. Alternatively, some isolates from VVC patients with low shedding might be in mixed VVC infections with several C. albicans strains, including a more virulent strain whose colony was not picked by simple chance. Further longitudinal studies of strains from asymptomatic individuals and/or those with RVVC and further depth in sampling of multiple isolates may help to resolve these potential complicating factors.

It will be of particular interest to understand if “pathogenic” phenotypes are more likely to be associated with negative outcomes in pregnancy or in recurrent VVC (71). However, it is not yet clear if these differences are conserved among all VVC pathogenic and colonizing strains nor whether these epithelial transcriptional differences translate into functional divergence. Furthermore, there are many other *Candida* epithelial cell-interacting factors and other differentially regulated epithelial pathways that may be just as important but remain untested in the context of this phenotype that differentiates VVC pathogenic strains. It is possible that *in vitro* assays to determine the pathogenic potential of clinical isolates would permit more antifungal treatments targeted toward pathogenic rather than colonizing strains. Furthermore, such assays would provide tools for identifying fungal virulence factors and epithelial responses that may be decisive in the switch from commensal to pathogen. Discovery of the molecular mechanisms underlying this phenotypic difference will therefore be of particular interest going forward.

## MATERIALS AND METHODS

### Microbial strains and culture conditions.

A total of 36 clinical C. albicans strains were employed in this study, in addition to the reference strain SC5314. The list of C. albicans clinical isolates is reported in Table S1C in Data Set S2 in the supplemental material. The core group of strains used throughout this study is a set of 16 clinical isolates obtained from women with VVC or healthy colonized women described by Pericolini et al. (19). All of the subjects were nondiabetic and were not taking antifungal therapeutics. Briefly, colonizing strains were obtained from women attending an obstetrics/gynecology clinic for routine screening, while VVC subjects had at least two diagnostic symptoms (vaginal discharge, itching, burning, or dyspareunia). None of the recruited women had RVVC. Further recruitment of subjects from the same populations yielded an additional group of 9 isolates from women with VVC (*n* = 7) or healthy colonized women (*n* = 2) that were used only for selected experiments and were initially described by Ardizzoni et al. (44). Details of the women from which these strains were obtained together with the ethics statements have been provided elsewhere (19, 44). A second cohort of isolates (project RC 13/18 ID Study 2715) was obtained through a different study at the IRCSS Burlo Garofolo of Trieste and collected from women with RVVC (*n* = 5) or healthy colonized (*n* = 5) women. Samples were obtained from asymptomatic women attending the gynecology clinic during screening programs or from women with a history of RVVC being seen for follow-up diagnosis and therapy. Details of these samples (screening for bacteria, hyphae, and neutrophils) are included in Table S1D in Data Set S2.

Fungal cultures were maintained by weekly passages onto Sabouraud dextrose agar (SDA) plates (Oxoid, Milan, Italy). The day before each experiment, fresh *Candida* cultures were seeded onto SDA plates or YPD (yeast extract-peptone-dextrose) broth according to the experimental protocol and incubated at 37°C. After overnight incubation, fungal cells were harvested by a sterile inoculating loop, suspended in phosphate-buffered saline (PBS) (EuroClone, Whethereby, United Kingdom), washed twice by centrifugation at 3,500 rpm for 10 min, counted with a Bürker chamber and suspended at the desired concentration in culture media different for every experiment (see below). The reference strain, Lactobacillus rhamnosus ATCC 7469, was maintained in frozen stocks at −80°C in tryptic soy broth (TSB) (Difco Laboratories, Detroit, MI, USA) with 5% (vol/vol) glycerol. Bacterial cultures were maintained by weekly passages onto De Man-Rogosa-Sharpe (MRS) agar (Oxoid, Milan, Italy) plates.

### Vaginal epithelium.

The human A431 vaginal epithelial cell line, obtained from ATCC, was grown in Dulbecco’s modified Eagle’s medium (DMEM) plus 10% defined fetal bovine serum (FBS) (HyClone, Logan, UT, USA), gentamicin (50 mg/mL) (Bio Whittaker, Verviers, Belgium), ciprofloxacin (Ciproxin) (2 mg/mL) (ICN), and l-glutamine (2 mM) (EuroClone, Milan, Italy).

One day before the experiment, vaginal cells (4 × 10^5^/mL/well) in DMEM plus 5% FBS were seeded in a 24-well plate and incubated for 24 h at 37°C plus 5% CO_2_ to allow the formation of a monolayer. For the quantification of C. albicans hyphal segment by protocol iii, cells were seeded in 8-well chamber slides.

### MLST analysis.

The isolates were tested by MLST as previously described (72, 73), using the consensus scheme available at https://pubmlst.org/organisms/candida-albicans. The ABC type (A, B, or C) and mating type-like (MTL) types (a/α, a/a, or α/α) were determined by PCR as previously described (74). Similarities between sequence data were analyzed in terms of *p*-distance with MEGA version 11 (75), and results with 500 bootstrap replications are depicted as a dendrogram by the unweighted-pair group method with arithmetic averages (UPGMA). The analyses were based on concatenated data from all known polymorphic sites, duplicated to allow the discrimination of homozygous and heterozygous differences (73).

### *ECE1* genotypic analysis.

DNA was isolated from each strain by Smash & Grab (76) and was amplified with the forward primer 5′-ATCATCCACCATGCTCCAG-3′ and either 251REV (5′-TTAGAGATAAGTCTTGGAGCATTAGC-3′) or 255REV (5′-TTGTTGAACAGTTTCCAGGACG-3′). Primers were designed within the P2 and P4 regions in areas most highly conserved in the available *ECE1* sequences from GenBank. PCR fragments were directly sequenced by Sanger sequencing. For amplifications that gave scrambled sequence indicating frameshifts, PCR products were cloned into pGEM-T Easy (Promega), several clones were sequenced by Sanger sequencing, and two consensus alleles were constructed. In some cases, frameshifts were outside the CL-encoding sequence, so both alleles shared the same CL sequence. Sequences were trimmed and then aligned with Clustal Omega (77), followed by nearest-neighbor joining in Simple Phylogeny (78).

### Quantification of C. albicans hyphal segments.

**(i) Protocol i.** A loop of each C. albicans strain was grown for 18 h at 37°C in 10 mL of RPMI 1640 under rotation. Cells were washed and resuspended in 100 μL of PBS (EuroClone, Wetherby, United Kingdom) plus 1% bovine serum albumin (BSA) (Sigma-Aldrich). Once washed, calcofluor white (1:20 from 100 μg/mL stock; Fluorescent Brightener 28 F3543) (Sigma-Aldrich) was added, and cells were incubated for 30 min on ice. Cells were washed three times and resuspended in 50 μL. A 10-μL aliquot was imaged with a Zeiss Axiobserver Z1 microscope and Axiocam MRm camera using DAPI (4′,6-diamidino-2-phenylindole) filter, using a Plan Neofluor 40-by-0.75 numerical aperture (NA) air objective, and Zeiss Axiovision software. z-stacks were processed in ImageJ by creating maximum projections and then scored as follows. Morphology was scored by counting all elongated cells, either pseudohyphae or hyphae, as filamentous. Calcofluor white staining was used to identify septa and thus to identify individual filament segments in hyphae. Counts were pooled for all fields imaged of a given sample.

**(ii) Protocol ii.** The day before each experiment, fresh *Candida* cultures were seeded onto SDA plates and incubated overnight at 37°C. Cells were harvested by a sterile inoculating loop, washed in PBS, and suspended at 4 × 10^6^ yeast cells/mL. The suspension was then divided into 3 aliquots, each of them was washed again, and the pellets were resuspended in PBS, in gRPMI (i.e., RPMI 1640 [Gibco, Grand Island, NY, USA] with 2 mM l-glutamine [EuroClone, Milan, Italy]) or in sgRPMI (i.e., RPMI 1640 with 2 mM l-glutamine and 10% defined FBS [Defined HyClone, Logan, UT, USA]). The day before each experiment, fresh L. rhamnosus cultures were seeded onto MRS plates and incubated overnight at 37°C in anaerobic conditions. Bacteria were harvested by a sterile inoculating loop, suspended in TSB, and washed with PBS. The bacterial concentration was calculated by interpolating optical density (OD) values to a stored *Lactobacillus* standard curve, which had been previously set up in our laboratory. The bacterial concentration was adjusted to 4 × 10^7^ lactobacilli/mL and divided into 2 aliquots. Each aliquot was pelleted and resuspended in gRPMI and sgRPMI. A combination of 100 μL of C. albicans suspension with or without L. rhamnosus in gRPMI or sgRPMI was then seeded in a Lab-Tek II chamber slide (Nalge Nunc International, Naperville, IL, USA). The chamber slide was incubated at 37°C with 5% CO_2_ for 3 h. At 15 min before the end of the incubation, 40 μL of 1% Uvitex 2B fluorescent dye (Polysciences, Inc., PA, USA) was added to each well. All the wells were then washed twice (5 min each) with gRPMI or sgRPMI (all kept at 37°C) and fixed for 30 min with 4% paraformaldehyde (PFA) (Sigma-Aldrich) in PBS at +4°C. The slides were then washed twice with cold PBS (10 min each). The chamber walls were removed, and the slide surface was treated with ProLong Gold antifade reagent (Molecular probes, Invitrogen, St. Louis, MO, USA). The cells were imaged by epifluorescence microscopy Nikon Eclipse 90i (Nikon Instruments, Tokyo, Japan). Five fields/well/experimental condition were imaged and counted for morphology, as described for protocol i. Hyphal segments were counted considering the septa separating one hyphal segment from another. Only fields containing a total number of fungi ranging between 50 and 200 were chosen.

**(iii) Protocol iii.** One hundred microliters of each C. albicans strain suspension was seeded in the wells of Lab-Tek II chamber slides containing a monolayer of A431 cells in DMEM plus 5% FBS. The chamber slide was incubated at 37°C with 5% CO_2_ for 24 h. After incubation, 40 μL of 1% Uvitex 2B was added to each well and the chamber slide was placed again at 37°C with 5% CO_2_ for a further 15 min. At the end of the incubation, all the wells were washed, fixed, and imaged by epifluorescence microscopy. Five fields/well/experimental condition were imaged and counted for morphology as described for protocol i. Only fields containing a total number of fungi ranging between 50 and 200 were chosen.

### Candidalysin proteomics.

Strains were grown in yeast nitrogen base (YNB) medium containing 2% sucrose, 75 mM 3-N-morpholinopropanesulfonic acid (MOPS) buffer (pH 7.2), and 5 mM *N*-acetyl-d-glucosamine at 37°C. After solid-phase extraction of hyphal supernatants, the resulting concentrated peptides were analyzed as described previously (37). Briefly, dried peptides were resolubilized in 100 μL of 0.2% HCOOH in 71:27:2 (vol/vol/vol) acetonitrile (ACN)-H_2_O-dimethyl sulfoxide (DMSO) and filtered (10-kDa molecular weight cutoff [MWCO]) at 14,000 × *g* for 15 min for analysis on a Thermo QExactive Plus with Ultimate 3000 nLC. Peptides were separated on an Accucore C_4_ column (15 cm by 75 μm, 2.6 μm) at 50°C. Gradient elution (A, 0.2% HCOOH in 95:5 H_2_O-DMSO; B, 0.2% HCOOH in 85:10:5 ACN-H_2_O-DMSO) was applied: 0 to 1.5 min at 60% B, 35 to 45 min at 96% B, and 45.1 to 60 min at 60% B. Cations generated at 2.2 kV (Nanospray Flex Ion Source) were measured in full MS/ddMS2 mode. The MS1 settings were as follows: *m/z* = 350 to 1,500, R = 70,000 (full width at half maximum [FWHM]), automatic gain control (AGC) target = 1 × 10^6^, ITmax = 200 ms. The top 15 precursors with an isolation width of *m/z* = 2 (*z* = 2 to 6) underwent high-energy collisional dissociation (HCD) fragmentation at 28% normalized collisional energy (NCE) (N2). MS2 spectra were monitored at the following settings: R = 17.5k, AGC target = 2 × 10^5^, and maximum number of allowed iterations (ITmax) = 150 ms. Spectra were searched against the Candida Genome Database of C. albicans SC5314 (10 October 2021) using Proteome Discoverer 2.4 with Sequest HT. FASTA files were manually modified in case of alternative *ECE1* alleles. Both unspecific and tryptic cleavages with dynamic Met oxidation were considered using precursor and fragment mass tolerances of 10 ppm and 0.02 Da, respectively. Target Decoy PSM Validator was used for *q* value validation of peptide spectral matches with a false-discovery rate (FDR) of <1%.

### Quantification of fungal growth in YPD broth.

To analyze total fungal growth of the different fungal strains in YPD medium, a loop of each C. albicans strain was grown for 24 h at 37°C in 5 mL of YPD broth under rotation. After incubation, fungal cell suspensions were centrifuged at 3,500 rpm for 5 min and cell pellets were resuspended in 3 mL of PBS and then counted with a Bürker counting chamber.

### Quantification of C. albicans shedding and growth on vaginal epithelium.

The shedding experiments were performed following 2 different protocols for shedding and exfoliation as described here and schematized in Fig. 5A. The shedding protocol was performed according to Graf et al. (54). Briefly, shedding of C. albicans strains was measured after 24 h of infection of an A431 monolayer (4 × 10^5^/mL/well) with the different C. albicans clinical isolates (4 × 10^5^/mL/well). Supernatants were collected and vortexed, whereas cells were treated with 0.2% Triton X-100 to lyse the host cells and release adherent fungal cells. One hundred microliters of supernatants or lysate samples were serially diluted in PBS and seeded in SDA plates. After 48 h of incubation at 37°C, CFU were counted. This protocol allowed us to determine the amount of live *Candida* cells in supernatants, adhered to epithelium and the total fungal growth (Fig. 5A, upper part, shedding protocol). In selected experiments, to analyze the role of type I interferon, the neutralizing anti-human IFNAR2 antibody (4 ng/mL) (PBL InterferonSource) was added to epithelial cells for 3 h before infection as described previously (63).

For the exfoliation protocol, shedding of C. albicans strains was measured after 24 h of infection of the A431 monolayer (4 × 10^5^/mL/well) with the different C. albicans clinical isolates (4 × 10^5^/mL/well). Supernatants were collected by gently pipetting up and down 5 times in each well to recover free fungal cells and fungi attached to or that penetrated the exfoliated epithelial cells. This supernatant was then centrifuged at 1,500 rpm for 5 min with a Microfuge 18 (Beckman Coulter) with an F241.5P rotor and washed twice with warm medium to remove free *Candida* in the supernatant, enriching for exfoliated or shed cells not in suspension. Cellular pellets were then treated with 0.2% Triton X-100 to lyse the shed host cells and release adherent/penetrating fungal cells. Then 100 μL of each cell suspension was serially diluted in PBS and spread in SDA plates. After 48 h of incubation at 37°C, CFU were counted. Confocal analysis of the exfoliated cell pellet was performed by resuspending the washed pellet (Fig. 5A; dark green) in 100 μL of PBS plus 1% bovine serum albumin (BSA) without Triton lysis (Fig. 5A; exfoliation protocol). Resuspended pellets were labeled with 20 μL of Uvitex 2B (1%) in PBS and 5(6)-carboxyfluorescein diacetate (CDFA) (which labels cytosolic proteins) (1 μL from a 54.3 μM stock solution) for 20 min on ice in the dark, washed, and resuspended in 100 μL of PBS plus 50% glycerol. Slides were imaged using a 63× Plan-Apo oil immersion objective mounted on a Leica SP8 confocal microscope equipped with 405-nm and white light lasers. Samples were excited using the 405-nm and 488-nm wavelengths for Uvitex 2B and CFDA, respectively. Mosaic images were acquired using the Navigator tool of LasX software.

### Adhesion assay.

Yeast cells were suspended in DMEM plus 5% FBS at 4 × 10^5^/mL. Then 100 μL of each cell suspension was seeded in triplicate in a 96-well microtiter plate. The plate was incubated for 2 h at 120 rpm and 37°C. After incubation, the wells were washed twice with PBS kept at room temperature to remove unattached yeasts. The adhesion of yeasts was quantified by means of the crystal violet (CV) protocol as previously described (79), with minor modifications. Briefly, 100 μL of 1% CV was added to each well and allowed to stain for 5 min. The wells were then washed twice with PBS at room temperature. Finally, 100 μL of 33% acetic acid was added to each well and absorbance was read at 570 nm.

### Quantification of C. albicans-induced damage of epithelial cells.

Lactate dehydrogenase (LDH) release from epithelial cells infected with *Candida* strains was measured after 24 h of infection of the A431 monolayer (4 × 10^5^/mL/well) with the different *Candida* clinical isolates (4 × 10^5^/mL/well at an MOI of 1:1 or 2 × 10^6^/mL/well at an MOI of 1:5) by a specific commercially available kit (BioVision) following the manufacturer’s instructions. Calculation of the percentage of cytotoxicity was performed as follows:

$$\text{\% cytotoxicity}=\frac{\left(\text{test sample}\;-\;\text{low control}\right)}{\left(\text{high control}\;-\;\text{low control}\right)}\times 100$$

### Determination of cytokine production.

IL-1β production from epithelial cells infected with *Candida* strains was measured after 24 h of infection of the A431 monolayer (4 × 10^5^/mL/well) with the different *Candida* clinical isolates (2 × 10^6^/mL/well at an MOI of 1:5) by a specific commercially available enzyme-linked immunosorbent assay (ELISA) kit (Thermo Fisher) following the manufacturer’s instructions.

### RNA sequencing and analysis.

For the RNA-seq analysis, a monolayer of A431 vaginal cells was infected with C. albicans CA01887 and CA14314 (MOI of 1:1) as described above. After 24 h of incubation at 37°C and 5% CO_2_, samples were collected in Eppendorf tubes and centrifuged at 3,500 rpm for 5 min. After centrifugation, supernatants were discarded and cell pellets were frozen at −80°C before shipment on dry ice. RNA preparation and next-generation sequencing (NGS) analysis were performed using a protocol based on the NEBNext Ultra II Directional RNA library prep kit (Illumina) and sequencing on a NovaSeq6000 (Illumina) at Eurofins (Eurofins, LLC). The RNA library preparation includes purification of poly(A)-containing mRNA molecules, mRNA fragmentation, random-primed cDNA synthesis (strand specific), Adapter ligation and adapter-specific PCR amplification. Using Galaxy (80), paired-end FASTQ files were analyzed for quality using FastQC and MultiQC (81). All experiments yielded reads that were of high quality according to FastQC, with *Candida*-epithelial cell mixtures having the expected lower GC content due to lower GC in fungal genes. The paired-end reads were trimmed with Trimmomatic (82) and then aligned to the human GRCh38 assembly using HISAT2 (83) in Galaxy. Next, read counts per gene in each sample were generated using HTSeq (84) and Ensembl (85) version 103 annotation of GRCh38 in Galaxy. Read counts per gene were analyzed using R/DESeq2 (86) to examine the correlation of read counts per gene across samples and test for differentially expressed genes. Genes expressed at a low level (those with fewer than 10 mapped reads across all samples) were removed from the analysis. Read counts of genes for duplicate samples were highly correlated by principal-component analysis. Next, levels of gene expression among duplicates of the CA01887 infection were compared to duplicates of uninfected epithelial cells; the same was done for the CA14314 infection, using R/DEseq2 and a false-discovery rate (FDR) of <0.01. The complete set of results from the R/DESeq2 analyses is available in Data Set S5 and Data Set S6. A total of 1,537 genes were differentially expressed with CA01887 infection and 1,234 genes with CA14314 infection. The two lists of differentially expressed genes, including the corresponding fold change and FDR for those genes, were analyzed using Ingenuity Pathway Analysis (Qiagen). A core analysis was performed, followed by a comparative analysis of canonical pathways. A full list of IPA-identified pathways is presented as Fig. S3 and is shown in Data Set S3. Differentially expressed genes were also analyzed for enriched GO biological process terms using PantherDB GO-Slim Biological Process Overrepresentation Analysis, and DAVID GO Biological Process FAT terms Enrichment Analysis (87–89). An FDR of <0.01 was used to create the list of differentially expressed genes from the R/DESeq2 results for comparisons of infected with CA01887 or CA14314 versus uninfected. An FDR of <0.05 was used for comparisons of CA01887 infected to CA14314 infected. For display of fold change of DESeq2 data in Fig. 6, any differences that were not statistically significant are shown in white, while upregulated genes are in yellow and downregulated genes are in blue. Data were plotted as log_2_ fold change with tools available at http://www.heatmapper.ca/expression/.

10.1128/mbio.00107-23.8 DATA SET S5 Full DESeq2 analysis for CA01887. Shown is a spreadsheet with the DESeq2 analyses for CA01887 performed as described in Materials and Methods. Download Data Set S5, XLSX file, 6.6 MB. Copyright © 2023 Sala et al. 2023 Sala et al. https://creativecommons.org/licenses/by/4.0/ This content is distributed under the terms of the Creative Commons Attribution 4.0 International license.

10.1128/mbio.00107-23.9 DATA SET S6 Full DESeq2 analysis for CA14314. Shown is a spreadsheet with the DESeq2 analyses for CA1431, performed as described in Materials and Methods. Download Data Set S6, XLSX file, 6.6 MB. Copyright © 2023 Sala et al. 2023 Sala et al. https://creativecommons.org/licenses/by/4.0/ This content is distributed under the terms of the Creative Commons Attribution 4.0 International license.

### Statistical analysis.

Statistical analyses were performed with GraphPad 8 (Prism). Quantitative variables were tested for normal distribution by Shapiro-Wilk test. Statistical differences between groups were assessed by the two-tailed Student's *t* test and one-way analysis of variance (ANOVA) followed by Tukey’s multiple-comparison test or by the nonparametric Mann-Whitney U test and Kruskal-Wallis test, followed by Dunn’s multiple-comparison test. The specific test used for each comparison is detailed in the corresponding figure legend. A *P* value of <0.05 was considered significant. Significance throughout the figures is indicated as follows: *, *P* < 0.05; **, *P* < 0.01; and ***, *P* < 0.001.

### Data availability.

Gene expression data and sequence data are accessible at the NCBI Gene Expression Omnibus under accession no. GSE207081.
