# Supplementary figures and images for: A New Phenotype in Candida-Epithelial Cell Interaction Distinguishes Colonization- versus Vulvovaginal Candidiasis-Associated Strains
Source: mBio. 2023 Mar 1;14(2):e00107-23. doi: 10.1128/mbio.00107-23 (PMC10128025; doi:10.1128/mbio.00107-23)

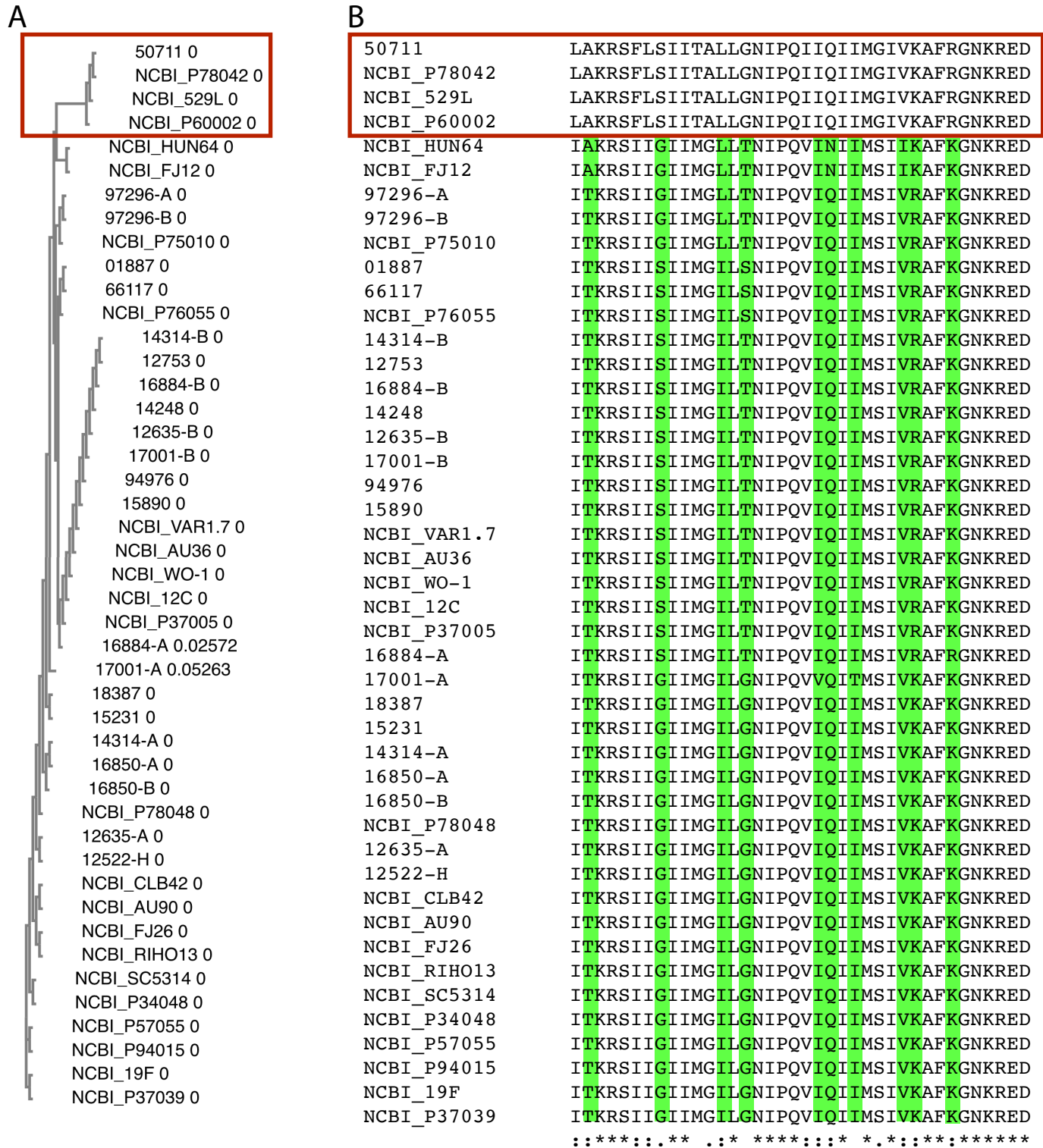

Figure S1. Sala et al.

Supplement: FIG S1 [file mbio.00107-23-s0001.pdf]

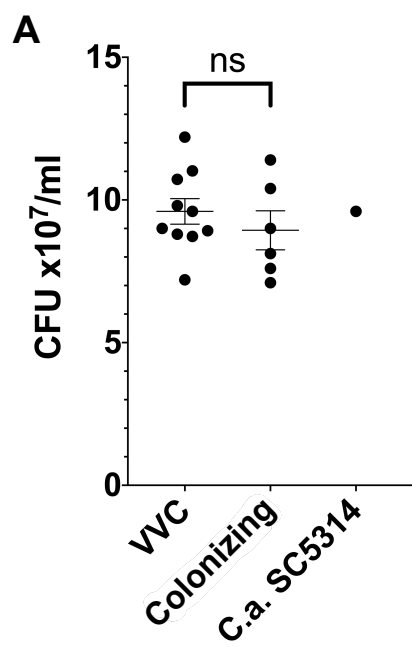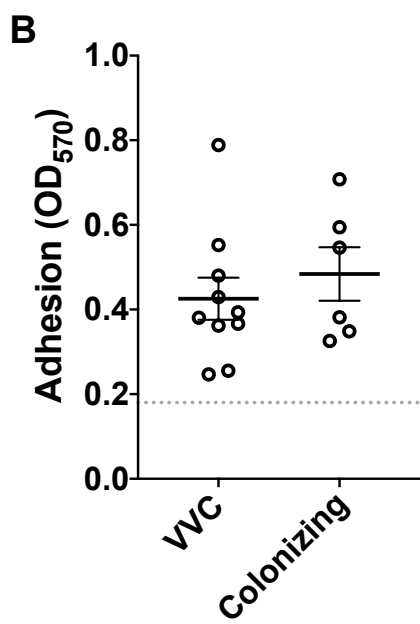

Supplement: FIG S2 [file mbio.00107-23-s0002.pdf]

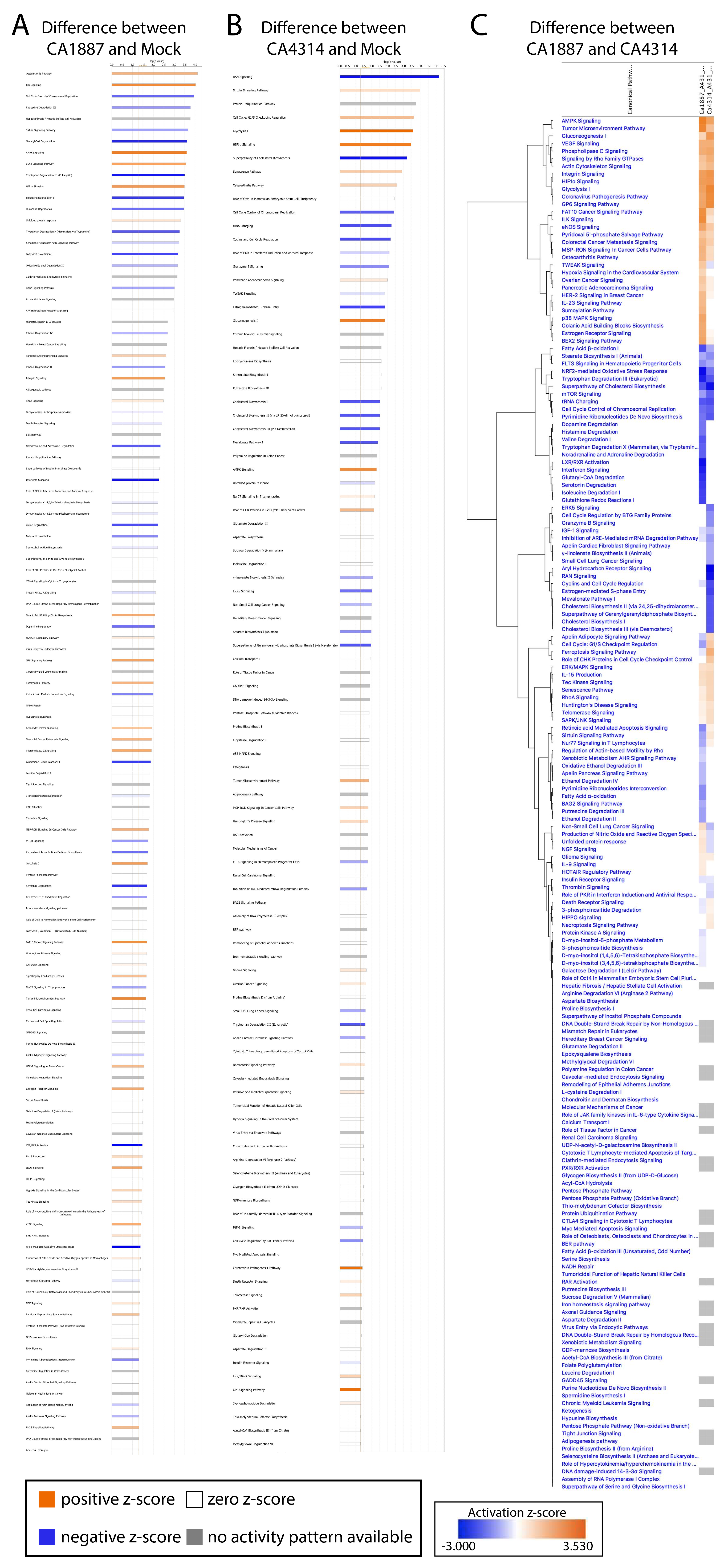

Supplement: FIG S3 [file mbio.00107-23-s0003.tif]
